# Characterisation of enterovirus RNA detected in the pancreas and other specimens of live patients with newly diagnosed type 1 diabetes in the DiViD study

**DOI:** 10.1007/s00125-021-05525-0

**Published:** 2021-08-14

**Authors:** Sami Oikarinen, Lars Krogvold, Bjørn Edwin, Trond Buanes, Olle Korsgren, Jutta E. Laiho, Maarit Oikarinen, Johnny Ludvigsson, Oskar Skog, Mahesh Anagandula, Gun Frisk, Heikki Hyöty, Knut Dahl-Jørgensen

**Affiliations:** 1 Faculty of Medicine and Health Technology, Tampere University, Tampere, Finland; 2 Paediatric Department, Oslo University Hospital, Oslo, Norway; 3 Faculty of Medicine, University of Oslo, Oslo, Norway; 4 The Intervention Centre, Department of HPB Surgery, Oslo University Hospital and Institute of Clinical Medicine, University of Oslo, Oslo, Norway; 5 Department of Hepato-Pancreato-Biliary Surgery, Institute of Clinical Medicine, Faculty of Medicine, University of Oslo, Oslo University Hospital, Oslo, Norway; 6 Division of Cancer, Surgery and Transplantation, Oslo University Hospital and Institute of Clinical Medicine, University of Oslo, Oslo, Norway; 7 Department of Immunology, Genetics and Pathology, Uppsala University, Uppsala, Sweden; 8 Crown Princess Victoria Children’s Hospital and Division of Pediatrics, Department of Biomedical and Clinical Sciences, Linköping University, Linköping, Sweden; 9 Fimlab Laboratories, Pirkanmaa Hospital District, Tampere, Finland

**Keywords:** Enterovirus, Laparoscopy, Organs, Pancreas, Persistent infection, RT-qPCR, Sequence, Type 1 diabetes, Viral RNA

## Abstract

**Aims/hypothesis:**

The Diabetes Virus Detection (DiViD) study is the first study to laparoscopically collect pancreatic tissue and purified pancreatic islets together with duodenal mucosa, serum, peripheral blood mononuclear cells (PBMCs) and stools from six live adult patients (age 24–35 years) with newly diagnosed type 1 diabetes. The presence of enterovirus (EV) in the pancreatic islets of these patients has previously been reported.

**Methods:**

In the present study we used reverse transcription quantitative real-time PCR (RT-qPCR) and sequencing to characterise EV genomes present in different tissues to understand the nature of infection in these individuals.

**Results:**

All six patients were found to be EV-positive by RT-qPCR in at least one of the tested sample types. Four patients were EV-positive in purified islet culture medium, three in PBMCs, one in duodenal biopsy and two in stool, while serum was EV-negative in all individuals. Sequencing the 5′ untranslated region of these EVs suggested that all but one belonged to enterovirus B species. One patient was EV-positive in all these sample types except for serum. Sequence analysis revealed that the virus strain present in the isolated islets of this patient was different from the strain found in other sample types. None of the islet-resident viruses could be isolated using EV-permissive cell lines.

**Conclusions/interpretation:**

EV RNA can be frequently detected in various tissues of patients with type 1 diabetes. At least in some patients, the EV strain in the pancreatic islets may represent a slowly replicating persisting virus.

**Graphical abstract:**

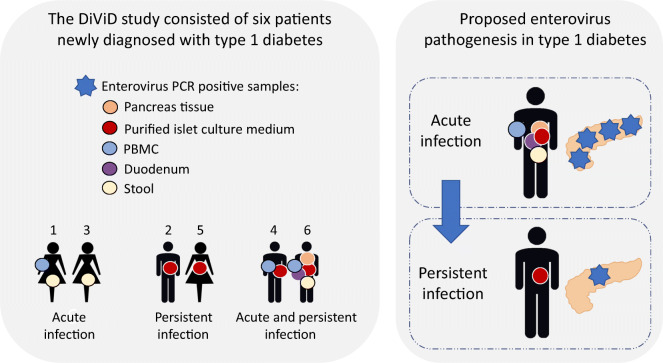

**Supplementary Information:**

The online version of this article (10.1007/s00125-021-05525-0) contains peer-reviewed but unedited supplementary material.

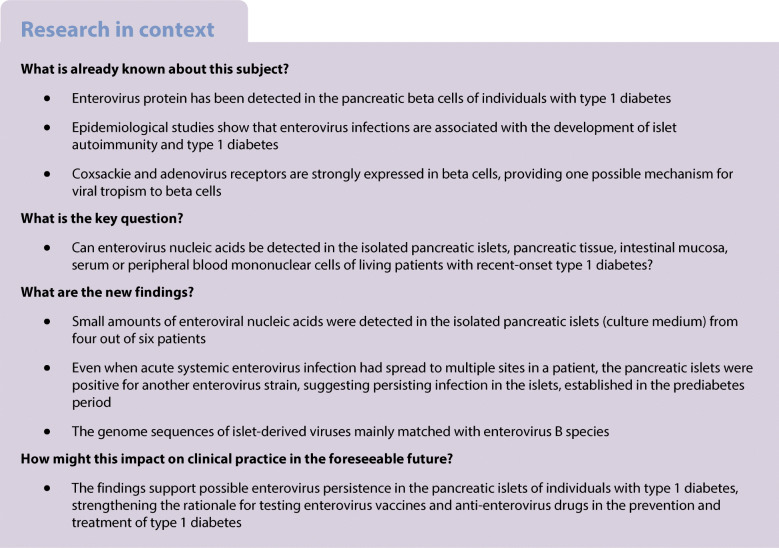



## Introduction

Type 1 diabetes is caused by the destruction of insulin-producing beta cells in the pancreas leading to insulin deficiency and lifelong dependency on daily insulin therapy. The risk of type 1 diabetes is determined by several genes, HLA genes being responsible for the majority of the genetic predisposition [1]. Immunological mechanisms are involved in the disease process, reflected by the presence of autoantibodies against beta cell proteins (islet autoantibodies) and by insulitis (inflammation of the pancreatic islets). However, the mechanisms of beta cell damage are not understood. The increasing incidence of type 1 diabetes, as well as studies among immigrants, suggest that external (environmental) factors play an important role in the pathogenesis [2].

Enterovirus (EV) infections are very common and are often subclinical. The virus replicates in the upper respiratory tract and in the intestinal mucosa; it often spreads to the blood and can reach internal organs including the pancreas. EVs are among the prime candidates for environmental triggers of type 1 diabetes. We have previously detected EVs in the pancreatic islets of all six patients with newly diagnosed type 1 diabetes in the Diabetes Virus Detection (DiViD) study from whom pancreatic tissue was collected 3–9 weeks after the diagnosis but in two of the nine control patients [3]. Both viral genome and viral capsid protein were reported and the results were confirmed in different laboratories. The small number of infected cells and low viral copy numbers found in reverse transcription quantitative real-time PCR (RT-qPCR) analysis possibly suggest a low-grade persistent-type infection in the islets. Such persisting EV infection has previously been linked to chronic cardiomyopathies where the virus is characterised by deletions in its genome, low rate of replication and abnormal balance between positive- and negative-stranded viral RNA in the infected tissue [4]. EVs have a tropism to pancreatic islets and insulin-producing beta cells. This has been documented in fatal EV infections, characterised by spreading of the virus to the islets where the virus causes cell damage and inflammation [5, 6]. In addition, human islets, and beta cells in particular, are permissive for EVs when infected in cell culture [7]. Insulin-producing beta cells express the coxsackie and adenovirus receptor (CAR), which serves as the major receptor for coxsackievirus B (CVB) group EVs, thus giving one possible biological explanation to this phenomenon [8, 9].

Indirect evidence from several case–control studies has suggested an association between EVs and type 1 diabetes, showing that EVs are found more frequently in individuals with type 1 diabetes than in those without diabetes [10]. The proportion of individuals with type 1 diabetes who are positive for EV protein in the pancreas is high (70–80%) and viral proteins are located mainly in insulin-positive cells [3, 11, 12]. Most of these studies report immunohistochemical detection of EV protein but in some studies the EV genome has also been found in islets using in situ hybridisation or RT-qPCR [3, 8, 12]. EV has also been successfully isolated from the pancreas of an individual with type 1 diabetes [5]. In addition, prospective studies have documented an excess of EV infections long before clinical type 1 diabetes is diagnosed, peaking before islet autoantibodies first appear, thus suggesting their possible role in initiating the processes involved in beta cell damage [13–15]. Recent studies have shown that among all human EV types (currently 116 EV A–D), the CVB groups show a risk association with type 1 diabetes [16, 17]. In addition, a very recent large-scale study in The Environmental Determinants of Diabetes in the Young (TEDDY) study, assessing all fecally shed viruses in relation to islet autoimmunity and type 1 diabetes, indicated that prolonged EV B infection may be associated with islet autoimmunity. Over 600 viruses were found but only EV Bs, and particularly CVBs, were indicated to have potential diabetogenic properties [18]. However, despite a variety of evidence linking EVs to type 1 diabetes, negative findings have also been published [19] and further studies are needed to obtain more information about a possible causal relationship.

One critical gap in knowledge is the lack of information on the nature of the EV infection observed in the pancreas of individuals with type 1 diabetes. The presence of EVs may represent an acute phase of the infection causing relatively rapid damage to the beta cells or a chronic low-grade infection driving long-term inflammation and immune-mediated beta cell damage, or both. The DiViD study is the first study to laparoscopically collect pancreatic tissue, together with duodenal mucosa, serum, peripheral blood mononuclear cells (PBMCs) and stools, from live patients with type 1 diabetes, thus allowing the analysis of the possible presence of EVs in multiple sites only weeks after the diabetes diagnosis. In our previous paper, the presence of EV in the pancreas of these patients was reported using RT-qPCR and immunohistochemistry [3]. In this study, we expand the previous findings by reporting further RT-qPCR results, detecting positive- and negative-strand EV genome in the pancreas, isolated pancreatic islet culture medium, duodenum, blood and stools of these patients, as well as performing sequence analyses characterising the virus strains present in these samples.

## Methods

### Patients and sample material

A total of six patients (three women, three men), aged 24–35 years (median 28 years), were recruited to the study (Table 1, ESM checklist). Pancreatic tail resection and duodenal biopsy samples, as well as serum, PBMC and stool samples, were collected 3–9 weeks (median 5 weeks) after the diagnosis of type 1 diabetes as previously described [20]. All tissue samples were processed for molecular techniques by rapid freezing in liquid nitrogen (snap-frozen aliquot) or by embedding in RNAlater (Qiagen, Hilden, Germany); all aliquots were stored at −80°C until analysed [20]. Blood samples were separated to plasma and PBMCs using Ficoll-Paque density gradient centrifugation and stored at −196°C. Stool samples were suspended (1:10) in Hanks’ medium and stored at −70°C until used for virus detection [21].

**Table 1:** Characteristics of the six patients included in the DiViD study

Patient	Age (years)	Sex (F/M)	BMI (kg/m^2^)	Time from diagnosis to biopsy (weeks)	HbA_1c_ at biopsy, (mmol/mol)	HbA_1c_ at biopsy (%)	Insulin (U kg^−1^ day^−1^)	Anti-GAD (<0.08 ai)^a^	Anti-insulin (<0.08 ai)^a^	Anti-ZnT8 (<0.12 ai) ^a^	Anti-IA2 (<0.10 ai) ^a^	HLA risk alleles (HLA DR3 and/or DR4)
1	25	F	21	4	50	6.7	0.5	1.76	0.7	0.28	0.16	Yes
2	24	M	20.9	3	89	10.3	0.35	0.79	<0.01	0.44	>3.00	Yes
3	34	F	23.7	9	54	7.1	0.17	1.77	<0.05	1.45	>3.00	Yes
4	31	M	25.6	5	57	7.4	0.4	0.77	0.1	<0.01	2.54	Yes
5	24	F	28.6	5	57	7.4	0.36	0.46	0.1	0.06	>3.00	Yes
6	35	M	26.7	5	54	7.1	0.52	1.85	<0.05	<0.01	< 0.04	Yes

^a^Arbitrary units according to Diabetes Antibody Standardization Program (DASP)

ai, auto antibody index; Anti-ZnT8, zinc transporter 8 autoantibody, Anti-IA2, islet antigen type 2 autoantibody; F, female; M, male

### Isolation and culture of pancreatic islets

The methodology of isolation of pancreatic islets was described in detail in our previous report [3] (Supplementary data). Briefly, the pancreatic duct was cannulated with a fine catheter for collagenase treatment. After digestion, cells were transferred to culture dishes and 300–700 islets from each individual were handpicked from the digested tissue under a dissecting microscope. Pancreatic islets were cultured for 5–6 days, and culture medium was collected for virus analyses on days 1, 3 and 5 post-isolation.

### RT-qPCR

In this study, the primary RT-qPCR analyses for snap-frozen pancreas tissue, homogenised pancreas in RNAlater, duodenum biopsies, culture supernatant fraction of isolated pancreatic islets, PBMCs, serum and stool samples (Table 2) were performed both in the Tampere laboratory (Tampere, Finland) and the Uppsala laboratory (Uppsala, Sweden). RNA extracted from islet cell culture medium, snap-frozen pancreatic tissue and stool samples were screened for EV in both laboratories, and for rhinovirus, norovirus, rotavirus and parechovirus in the Tampere laboratory. The PBMCs, duodenal biopsy and pancreas samples stored in RNAlater were screened for EV and rhinovirus only. RNA extractions and RT-qPCR methods are described in detail in our previous report [3] (Supplementary data).

**Table 2:** Summary of EV RT-qPCR results

Patient	Purified islets^a^	Pancreas		PBMCs	Serum	Duodenal biopsy	Stool
		Snap-frozen tissue	RNAlater tissue				
1	Neg	Neg	No sample	POS	Neg	Neg	POS
2	<10^b^	Neg	Neg	Neg	Neg	Neg	Neg
3	Neg	Neg	Neg	Neg	Neg	Neg	10^6^
4	<10^b^	Neg	Neg	POS	Neg	Neg	No sample
5	20^b^	Neg	Neg	Neg	Neg	Neg	Neg
6	<10^b^	<10^b^	40^b^	<10^b^	Neg	10^2b^	10^5^

The positivity is indicated as copy number of the enteroviral RNA (RT-qPCR, Tampere) or as the presence of EV-specific sequence (semi-nested RT-PCR, Uppsala). Genotyping of the EV was successful only from patient 3 stool sample and from all patient 6 samples (with the exception of purified islets where the genotype was different from the other sample types)

^a^Virus-positive in any of the culture media of purified pancreatic islets, collected on culturing days 1, 3 and 5

^b^Samples EV-positive in both of the two independent RT-qPCR methods

Neg, negative; Pos, positive

In addition, in the Tampere laboratory the presence of negative- and positive-strand EV RNA was tested using primers specific for either negative- or positive-strand RNA in the cDNA synthesis, followed by quantitative PCR reaction with both primers. The reverse transcriptase reaction (total volume of 20 μl) contained 5 μl of extracted RNA, RT buffer (Promega, Madison, WI, USA), 0.5 mmol/l deoxynucleotide triphosphates (Pharmacia Biotech, Uppsala, Sweden), 4 U of RNase inhibitor (Promega), 50 pmol of the negative-strand (4−) primer designed to 555–566 nucleotides of virus sequence in GenBank with accession no. AY752944 (4−) or the positive-strand primer (636+) nucleotide positions 452–471, and 20 U of Moloney murine leukaemia virus reverse transcriptase enzyme (Promega). The reverse transcriptase reactions were incubated for 60 min at 37°C.

Possible deletion in the 5′ untranslated region (5′UTR) termini of the EV genome was tested using earlier published primers [5]. Overlapping forward primers were targeted 1–79 nucleotides inward from the 5′UTR termini and reverse primer (E3Sub) was designed to nucleotide region 549–535 (positions refers to virus sequence in GenBank with accession no. AY752944) [4].

### Sequencing

EV-positive RT-qPCR products were sequenced covering a part (439–512 nucleotides) of the 5′UTR or a part of the viral protein 1 (VP1) coding region [22]. Sequencing reactions were carried out at the core facility at Uppsala University or using commercial sequencing service (Macrogen, Korea). The sequences were edited using Geneious 11.1.3 (https://www.geneious.com). Phylogenetic trees were created using a neighbour-joining method with 1000 bootstrap replicates (PHYLIP — Phylogeny Inference Package, Version 3.2, https://evolution.genetics.washington.edu/phylip.html) [23].

### Virus isolation

Virus isolation was carried out in the Tampere laboratory for all RT-qPCR EV-positive stool samples and islet cell culture medium samples using three cell lines (A549, CaCo-2 and HeLa from ATCC), which can be infected by different EVs. The cell lines were tested negative for mycoplasma using VenorGeM Classic Mycoplasma Detection kit (Minerva Biolabs, Berlin, Germany). The cells were inoculated with 0.2 ml of 10% stool suspension (Hanks’ medium supplemented with 0.2% BSA, 2.5 μg/ml amphotericin B, 2 5 μg/ml vancomycin, 20 μg/ml gentamicin and 32 μg/ml cefuroxime) and incubated at 37°C according to standard procedures [21]. The cytopathic effect was monitored and positivity was confirmed with EV-specific RT-qPCR and sequencing methods.

### Ethics

The DiViD study was approved by The Norwegian Governments Regional Ethics Committee. Written informed consent was obtained from all patients.

## Results

All six patients were found to be EV-positive by RT-qPCR in at least one of the tested sample types. The most frequently EV-positive sample type was isolated islet culture medium, in which four of the six patients were EV-positive. In contrast, the serum samples of all patients were EV-negative.

**Fig. 1:**
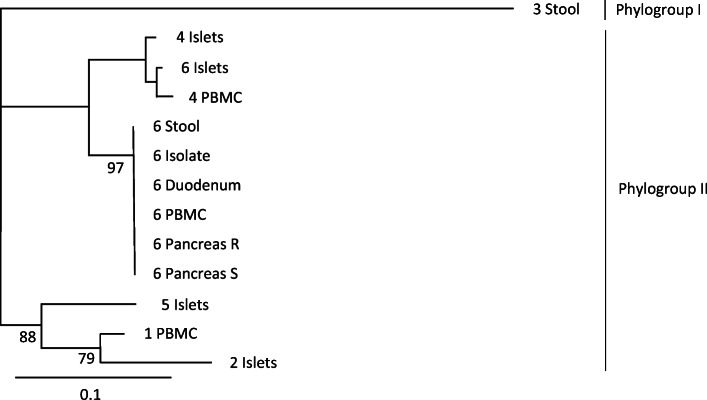
Phylogenetic analysis of EV-positive samples based on the partial nucleotide sequence of the 5′UTR region (439–512 nucleotides in E30 GenBank accession no. KP266571). EVs can be divided into phylogenetic groups I and II using nucleotide sequence of this genome region. Virus obtained from the stool sample of patient 3 clusters in phylogroup I and all other viruses to phylogroup II. The same EV nucleotide sequence was present in all EV-positive samples from patient 6 except for the islet culture medium, which contained a different viral nucleotide sequence. Patients are identified by numbers 1–6, followed by the type of sample sequenced. Bootstrap values over 75 are marked in the phylogenetic tree. The distance scale is shown in the lower left corner. Islets, medium of cultured islets; Isolate, isolated virus; Pancreas R, RNAlater sample from pancreas; Pancreas S, snap-frozen sample from pancreas

**Fig. 2:**
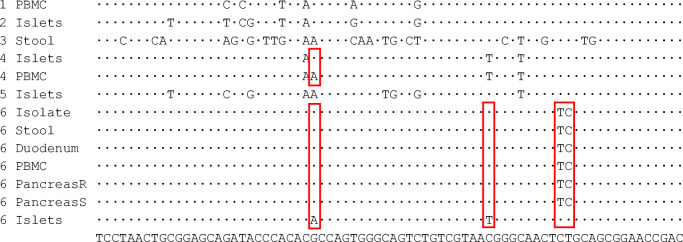
Sequence alignments of enterovirus strains detected in samples from patients with type 1 diabetes representing nucleotides 439–512 of the 5′UTR of E30 viral genome (GenBank accession no. AM237033). Patients are identified by numbers 1–6. Nucleotides that show variation to the consensus sequence (shown at the bottom of the figure) between virus strains are shown. Variable nucleotides between virus strains obtained from the same individual are marked with red rectangles. Islets, medium of cultured islets; Isolate, isolated virus; Pancreas R, RNAlater sample from pancreas; Pancreas S, snap-frozen sample from pancreas

At an individual level, patient 1 was EV-positive in PBMCs and stool but VP1 sequencing of the virus genome was not successful and the genotype could not be identified (Table 2). Patients 2 and 5 were EV-positive in the purified islet culture medium but EV-negative in all other samples. Patient 3 was EV-negative in all sample types except for stool, where the virus was typed as coxsackievirus A22 (CVA22) by VP1 sequencing. Patient 4 was EV-positive in the purified islet culture medium and in PBMCs. No stool sample was available from patient 4. Patient 6, on the other hand, was EV-positive in all sample types except for serum. The same 5′UTR sequence was present in this patient’s PBMCs, pancreatic tissue (in both frozen and RNAlater samples), duodenal biopsy and stool samples, suggesting the presence of the same virus strain in all these specimens. Based on the partial VP1 sequence detected in stool from this patient, this virus was typed as echovirus 30 (E30) (Fig. 1). However, a different EV strain was present in the purified islet culture medium, as the amplified product differed by four nucleotides from the E30 strain that showed identical 5′UTR sequence in all other specimens (a 74-nucleotide-long fragment of the 5′UTR of the viral genome successfully sequenced from all these sample types; Fig. 2 and Table 3). E30 was isolated from patient 6’s stool sample and replicated well in A549 cells but not in other cell lines (data not shown). All islet cell culture medium samples were negative for virus isolation.

**Table 3:** The genotyping results of the enterovirus-positive samples

Patient	Sample type	VP1 similarity (%)	5′UTR similarity (%)
1	PBMC		CVB3 (100.0)
2	Purified islets		CVB5 (97.0)
3	Stool	CVA22 (98.5)	EV B, EV C (100.0)
4	Purified islets		CVB3, E7, E13, E25, B83 (98.7)
4	PBMC		CVB3, CVB5, E3, E9, E11, E24, CVA9 (98.7)
5	Purified islets		CVB4 (98.7)
6	Purified islets		CVB3, E5, E9, E11 (100.0)
6	PBMC		E30 (100.0)
6	Pancreas S		E30 (100.0)
6	Pancreas R		E30 (100.0)
6	Stool	E30 (98.8)	E30 (100.0)
6	Duodenum		E30 (100.0)

All sequences were blasted against GenBank sequences and the best matches and similarity (%) with GenBank enterovirus types are presented

Pancreas R, pancreas tissue in RNAlater; Pancreas S, snap-frozen pancreas tissue

Based on amplification in RT-qPCR, the viral titres were generally quite high in stools and duodenal biopsies but much lower in PBMCs as well as in the frozen pancreatic tissue and culture medium from purified pancreatic islets (Table 2). All patients were negative for rhinovirus, parechovirus, rotavirus and norovirus in all tested sample types. Patient 3 had symptoms of an acute respiratory infection 2 weeks before the type 1 diabetes diagnosis while none of the other patients reported symptoms of infection during the past 3 months.

All detected EVs were genotyped using part of the 5′UTR nucleotide sequence. Combining these results with VP1 sequences, two of these viruses had a perfect match with the wild-type viruses in GenBank, and six had not been published earlier. The best match for these sequences were CVBs or echoviruses representing EV B species, except for the virus from the stool sample of patient 3, which was genotyped as CVA22 (EV C species) (Table 3).

The presence of positive- and negative-strand EV RNA genome was analysed using primers specific for either negative-strand or positive-strand RNA, or both, in the cDNA synthesis. EV-positive stool and duodenal samples showed positivity only for positive-strand RNA while the supernatant fraction from cultured isolated islets was EV-positive only when cDNA synthesis was performed with both primers (Table 4). Unfortunately, RNA from other sample types was not available for the analyses of positive- and negative-strand viral RNA.

**Table 4:** Nature of EV infection in samples

Patient	Sample type	RT-qPCR	Strand-specific RT-qPCR	
		Both positive and negative strands	Negative strand	Positive strand
3	Stool	Pos	Neg	Pos
4	Purified islets^a^	Pos	Neg	Neg
6	Purified islets^a^	Pos	Neg	Neg
6	PBMC	Pos	Neg	Neg
6	Duodenal biopsy	Pos	Neg	Pos
6	Stool	Pos	Neg	Pos

Positive- and negative-strand-specific RT-qPCR was performed. Based on the results, the stool and duodenal samples had indications of an acute infection, seen as the positive results on positive-strand RT-qPCR. Purified islets and PBMC samples were positive only when both primers were present in cDNA reaction

^a^Virus-positive in any of the culture media of purified pancreatic islets

Neg, negative; Pos, positive

All RT-PCR reactions to test the possible deletion in 5′UTR termini of the EV genome were negative, possibly indicating evidence for terminal deletions in the detected viruses.

## Discussion

The DiViD study is the first study where the presence of EVs in the pancreas, duodenum, stool and blood of living patients with recent-onset type 1 diabetes has been examined using sensitive molecular technologies. This unique design allowed us to study further the nature of EV infection previously reported in the pancreas of these patients [3]. Our results suggest that some individuals with type 1 diabetes are affected by a systemic EV B infection that has spread to the pancreas, as represented by patient 6 in this series. In addition, we found that patients 2, 4, 5 and 6 had evidence of a low-grade EV infection in the pancreatic islets without signs of systemic spread of the virus. These islet-resident viruses may have been present in a double-stranded form, as suggested by the detection of the EV genome only when both positive and negative stands of viral RNA were amplified in the same RT-qPCR reaction. In addition, their titres were very low which fits with persistent-type infection. Altogether, these observations could indicate a chronic infection in endocrine cells in which the virus may persist in low titres long after the acute phase of the infection. This finding not only supports the findings of previous studies where EV protein was present in the pancreatic islets of most individuals with recent-onset type 1 diabetes and where the number of infected islets and beta cells was low [11]. Our findings may also be in accordance with the recent discovery of the involvement of prolonged EV B infection in islet autoimmunity [18]. Such prolonged shedding of EV B may reflect either virus- or host-related factors that favour the development of EV B persistency.

In addition to pancreatic islets, patient 6 was also EV-positive in all other sample types except for serum. Sequence analyses confirmed the presence of the same E30 strain in the stool, duodenal biopsy, PBMCs and pancreas (in both frozen and RNAlater-treated pancreatic samples), suggesting an acute systemic infection in this patient. This was also supported by the fact that an infective E30 was isolated from the stool and it replicated well in A549 cells. Primary replication of EVs takes place in the lymphoid tissues of the oropharynx and in the intestinal mucosa. We have previously reported that individuals with type 1 diabetes are more frequently positive for EVs in the duodenal mucosa (endoscopic biopsy samples) [24]. The detection of EV in the duodenal biopsy in patient 6 is well in line with these previous findings.

Viral copy numbers in stool samples of patients 3 and 6 were high but only positive-strand EV RNA was detected, suggesting that the viral RNA originates from acute infection and intact virions. Patient 6 was the only one positive for EV in the duodenum and again only positive-strand RNA was detected. In theory, negative-strand RNA could also be present since it is synthesised during virus replication inside the infected duodenal cells. However, it could have remained undetectable due to the relatively low amount of the virus and the fact that in acute infection negative-strand genome is produced in much smaller amounts than positive-strand genome.

In patient 6, the viral titres were very low in PBMCs and pancreas, and the serum sample was completely EV-negative. This suggests that the peak of virus replication and a possible viraemia had occurred earlier. Altogether, these findings suggest that patient 6 had an active E30 infection that had probably started some time prior to the sample collection and that had also spread to the pancreas. This patient displayed no symptoms of an acute or recent infection.

Interestingly, the EV strain found in the pancreatic islet culture medium from patient 6 differed in four nucleotide positions (out of the 74 nucleotides of the amplified sequences) from the strain found in all other sample types including the whole pancreas tissue. The difference of four nucleotides strongly suggests the presence of another virus strain in the islets, possibly originating from a pre-existing persisting infection. For example, in our recent study, even 13 months of continuous replication of CVB during persistent infections in a pancreatic cell line did not produce mutations in this sequence [25]. Persistent EV infection has been shown to render pancreatic cells resistant to EV superinfection [26, 27] and, theoretically, this could also explain the lack of E30 in the isolated islets. In addition, patients 2 and 5 were EV-positive in the purified islet culture medium but EV-negative in all other sample types (including intact pancreatic tissue), suggesting possible EV persistence in the islets. Islet culture medium was also EV-positive only when both negative- and positive-strand primers were used in cDNA reaction. This fits with EV persistency where the amount of positive-stranded RNA is decreased and viral RNA is present in a double-stranded form [28]. EVs are released from infected cells to the cell culture medium during virus replication and usually viral titres are high after a few days’ culture. Importantly, in patient 6, EV was only detected in islet culture supernatant fractions on days 1 and 3 after isolation whereas on day 5 all supernatant fractions were negative, suggesting that the virus did not replicate efficiently in the islets ex vivo. It is possible that this EV is a slowly replicating or dormant virus that started to replicate when the islets were cultured in the absence of physiological signals from the surrounding tissue (e.g. type 1 IFNs, which are overexpressed in the pancreas of type 1 diabetes patients and can make the cells resistant to infection). In addition, we were not able to isolate infective virus from the islet culture medium. Altogether, these findings would fit with a persistent EV infection by a replication-incompetent virus strain [29]. It can be hypothesised that the acute E30 infection had precipitated the symptoms of type 1 diabetes in patient 6 while the persisting islet infection could have caused pre-existing beta cell damage. Patient 6 was positive for only one autoantibody (to GAD), suggesting that the autoimmune component could have been weaker than in the other patients.

The hypothesis wherein a chronic, persistent infection of pancreatic islets plays a role in the pathogenesis of type 1 diabetes is supported by previous studies showing that a high proportion of individuals with type 1 diabetes are positive for EV within the islets [3, 11, 30]. In addition, EVs are known to cause persistent infections in mice [29, 31] and in pancreatic ductal cell line PANC-1 [32]. Persistent EV infections are also reported in humans; in the myocardial tissue, such infection can cause chronic dilated cardiomyopathy [33] and persistent EV infections also occur in immunocompromised patients [34, 35]. EV persistence has been linked to deletions in the viral genome that occur in the 5′UTR of the viral genome, reducing its replication to such a low level that it may be difficult to detect without highly sensitive assays [29]. The viruses detected in this study could not be amplified using 5′UTR primers that cover this deleted genome region, suggesting that they may carry such deletion [4]. However, since we do not know the complete 5′UTR sequence of these viruses, it is possible that the negative result was due to primer mismatches leading to weaker amplification compared with our screening RT-qPCR using primers matching to practically all EV types. It is also possible that the 5′UTR of non-capsidated persisting EVs has been partly degraded by pancreatic enzymes, as shown in our recent work [36].

Patient 3 had an acute CVA22 infection based on the virus detection and molecular genotyping from the stool. This virus belongs to the EV C species and typically causes herpangina and central nervous system infections but so far it has not been connected to type 1 diabetes. Two weeks prior to diabetes diagnosis, patient 3 had upper respiratory tract symptoms, which could fit with the possible symptoms of CVA22. Patient 1 had EV in stools and PBMCs. However, possibly due to low viral titres, RT-PCR in the VP1 region was negative and the strain could not be genotyped. This patient had no clinical signs of viral infection prior to the diagnosis of type 1 diabetes.

Prospective studies have shown an excess of EV infections prior to islet autoantibody seroconversion, suggesting their possible role in the initiation of this process [13, 16]. On the other hand, case–control studies have indicated an excess of EVs in the blood of individuals newly diagnosed with type 1 diabetes, possibly reflecting the role of EV in later stages of the process and precipitation of the clinical disease [10]. EV infection can also accelerate the progression of beta cell-damaging autoimmune processes in NOD mice [37]. Thus, it is possible that if an EV infection spreads to the pancreas in an individual with ongoing beta cell damage, it can cause additional cell damage either directly or by immune-mediated mechanisms and precipitate the symptoms of type 1 diabetes [38]. Patient 6 could represent such a scenario, in which a low-grade persistent infection in the islets is followed by an E30 infection spreading to the pancreas. Previously E30 has been connected to the induction of islet autoimmunity [39] even though a recent large prospective study did not find such association [16].

Species B EVs, including CVBs and certain echoviruses, have been linked to type 1 diabetes [16, 17, 40, 41]. All 5′UTR sequences detected in the patients matched to CVBs and echoviruses, except for the CVA22 detected in the stool sample of patient 3. This predominance of species B EVs in the pancreas and PBMC samples is different from the predominance of species A EVs generally observed in stool samples [42]. Species B EVs, especially CVBs, have previously been linked to invasive and severe infections. Interestingly, the EV sequence detected in the islets isolated from patient 6 matched to an echovirus 5 (GenBank accession no. AF188359), which had persisted for 7 years in a patient with agammaglobulinaemia [35].

This study has certain limitations that need to be considered when interpreting the results. First, pancreatic tissue was analysed only at one time point and only six patients were included, making it impossible to generalise the findings to all individuals with type 1 diabetes. RT-qPCR, which was used in this study, has been used in several previous publications and has been reported to be highly sensitive in external quality control rounds (e.g. got best scores in Quality Control for Molecular Diagnostics rounds). However, the high sensitivity makes this method vulnerable to environmental contamination during sampling or in the laboratory during various steps of sample processing, which might lead to false-positive findings. However, even if we cannot exclude this possibility, we consider such contamination unlikely. The samples were collected in the operation room and were processed in a laboratory that did not handle viruses, all other virus RT-qPCRs that were performed were virus-negative, RT-qPCR controls were negative and, finally, the sequences of the amplified viruses originated from different virus strains. Moreover, six of the virus sequences had not been published earlier in GenBank and two sequences matched with GenBank wild-type strains. In addition, even though islet isolation was carried out in a single laboratory, the RT-qPCR and sequencing results were confirmed in two independent laboratories. It should also be noted that virus analyses of isolated pancreatic islets were carried out using islet cell culture medium while the isolated islet cells were not available for testing the presence of viral RNA.

In conclusion, these results confirm the findings from previous studies of the presence of EV genome in the pancreatic islets of some individuals with type 1 diabetes [3, 8]. The finding of two different EV strains simultaneously present in the pancreatic islets vs other tissues from the same patient corresponds with a relapse–remitting model for the development of type 1 diabetes wherein serial EV infections can cause cumulative insults to beta cells and viral persistence may play a role.

## Supplementary Information

ESM (PDF 74 kb)

## Data Availability

The primary data are available upon request from the corresponding author.

## Abbreviations

CAR: Coxsackie and adenovirus receptor
CVA22: Coxsackievirus A22
CVB: Coxsackievirus B
DiViD: Diabetes Virus Detection
E30: Echovirus 30
EV: Enterovirus
PBMC: Peripheral blood mononuclear cell
PEVNET: Persistent Virus Infection in Diabetes Network
RT-qPCR: Reverse transcription quantitative real-time PCR
5′UTR: 5′ Untranslated region
VP1: Viral protein 1
