# Supplementary material for: Characterisation of enterovirus RNA detected in the pancreas and other specimens of live patients with newly diagnosed type 1 diabetes in the DiViD study
Source: Diabetologia. 2021 Aug 14;64(11):2491–501. doi: 10.1007/s00125-021-05525-0 (PMC8494699; doi:10.1007/s00125-021-05525-0)
Supplement: Supplementary file 1 — (PDF 74 kb) [file 125_2021_5525_MOESM1_ESM.pdf]

Diabetologia

Checklist for reporting human islet preparations used in research

Adapted from Hart NJ, Powers AC (2018) Progress, challenges, and suggestions for using human islets to understand islet biology and human diabetes. Diabetologia https://doi.org/10.1007/s00125-018-4772-2

Islet preparation	1	2	3	4	5	6	7	8[a]
**MANDATORY INFORMATION**								
Unique identifier	1	2	3	4	5	6		
Donor age (years)	25	24	34	31	24	35		
Donor sex (M/F)	F	M	F	M	F	M		
Donor BMI (kg/m^2)	21	20.9	23.7	25.6	28.6	26.7		
Donor HbA_{1c} (mmol/mol) or other measure of blood glucose control	50	89	54	57	54	54		
Origin/source of islets[b]	the Nordic Network for islet transplantation	the Nordic Network for islet transplantation	the Nordic Network for islet transplantation	the Nordic Network for islet transplantation	the Nordic Network for islet transplantation	the Nordic Network for islet transplantation		
Islet isolation centre	Department of Immunology, Genetics and Pathology, Uppsala University, Uppsala, Sweden	Department of Immunology, Genetics and Pathology, Uppsala University, Uppsala, Sweden	Department of Immunology, Genetics and Pathology, Uppsala University, Uppsala, Sweden	Department of Immunology, Genetics and Pathology, Uppsala University, Uppsala, Sweden	Department of Immunology, Genetics and Pathology, Uppsala University, Uppsala, Sweden	Department of Immunology, Genetics and Pathology, Uppsala University, Uppsala, Sweden		
Donor history of diabetes? Please select yes/no from drop down list	Yes	Yes	Yes	Yes	Yes	Yes		
**If Yes, complete the next two lines if this information is available**								
Diabetes duration (years)	0.1	0.1	0.2	0.1	0.1	0.1		
Glucose-lowering therapy at time of death[c]								

RECOMMENDED INFORMATION								
Donor cause of death								
Warm ischaemia time (h)								
Cold ischaemia time (h)								
Estimated purity (%)								
Estimated viability (%)								
Total culture time (h)[d]								
Glucose-stimulated insulin secretion or other functional measurement[e]								
Handpicked to purity? Please select yes/no from drop down list								
Additional notes	The islet were isolated from living patients with T1D in the DiViD study	The islet were isolated from living patients with T1D in the DiViD study	The islet were isolated from living patients with T1D in the DiViD study	The islet were isolated from living patients with T1D in the DiViD study	The islet were isolated from living patients with T1D in the DiViD study	The islet were isolated from living patients with T1D in the DiViD study		

[a]If you have used more than eight islet preparations, please complete additional forms as necessary
[b]For example, IIDP, ECIT, Alberta IsletCore
[c]Please specify the therapy/therapies
[d]Time of islet culture at the isolation centre, during shipment and at the receiving laboratory
[e]Please specify the test and the results
